# Spatial modelling of the tumor microenvironment from multiplex immunofluorescence images: methods and applications

**DOI:** 10.3389/fimmu.2023.1288802

**Published:** 2023-12-20

**Authors:** Gayatri Kumar, Renganayaki Krishna Pandurengan, Edwin Roger Parra, Kasthuri Kannan, Cara Haymaker

**Affiliations:** Department of Translational Molecular Pathology, MD Anderson Cancer Center, Houston, TX, United States

**Keywords:** complete spatial randomness, spatial clustering, multiplex immunofluorescence, tumor microenvironment, multiplexed imaging and cellular neighborhood

## Abstract

Spatial modelling methods have gained prominence with developments in high throughput imaging platforms. Multiplex immunofluorescence (mIF) provides the scope to examine interactions between tumor and immune compartment at single cell resolution using a panel of antibodies that can be chosen based on the cancer type or the clinical interest of the study. The markers can be used to identify the phenotypes and to examine cellular interactions at global and local scales. Several translational studies rely on key understanding of the tumor microenvironment (TME) to identify drivers of immune response in immunotherapy based clinical trials. To improve the success of ongoing trials, a number of retrospective approaches can be adopted to understand differences in response, recurrence and progression by examining the patient’s TME from tissue samples obtained at baseline and at various time points along the treatment. The multiplex immunofluorescence (mIF) technique provides insight on patient specific cell populations and their relative spatial distribution as qualitative measures of a favorable treatment outcome. Spatial analysis of these images provides an understanding of the intratumoral heterogeneity and clustering among cell populations in the TME. A number of mathematical models, which establish clustering as a measure of deviation from complete spatial randomness, can be applied to the mIF images represented as spatial point patterns. These mathematical models, developed for landscape ecology and geographic information studies, can be applied to the TME after careful consideration of the tumor type (cold vs. hot) and the tumor immune landscape. The spatial modelling of mIF images can show observable engagement of T cells expressing immune checkpoint molecules and this can then be correlated with single-cell RNA sequencing data.

## Introduction

Identifying the spatial interactions in the tumor microenvironment (TME) that mitigate positive immune response to treatment are of incomparable importance for improving the success of clinical trials (1, 2). It is also important to understand the differences in immune-tumor interactions to make an informed decision on patient selection and on the inclusion in checkpoint therapy and trials. The TME also gives a snapshot of the cells in their natural biological state (3, 4). The spatial relationships obtained from multiple regions of interest (ROIs) of patient samples can recapitulate the cellular milieu and the intratumoral heterogeneity (5–7). The higher density of different immune populations in the TME and effector T cell interactions are indicators of immune response (8, 9). While the abundance of cytotoxic T lymphocytes itself is a sufficient indicator of immune response in a number of cancers, organ level differences can be observed in immune infiltration in other cancers (10). Spatially resolving the diverse cellular features that constitute the complex tumor landscape is important in identifying the cell states and geographic diversity of cell types and their clinical consequences (11, 12). Immune infiltration can be quantified using relevant spatial mathematical functions that provide an unambiguous distinction between a random distribution and a clustered population (13, 14). The application of spatial mathematical models in identifying second-order effects between different features in data is well studied in ecology (15, 16). These robust models can be applied to the TME to measure infiltration using different immune cell types and malignant cells as features or marks. The application of spatial models such as the Gibbs hardcore process has been shown to identify loss of heterotypic contact-inhibition of locomotion among cancer cells, which is a natural part of the spatial birth and death process (17). The application of ‘pair correlation function’ from the geospatial toolbox spatstat (18) on the tissue microarrays (TMAs) in Diffuse Large B-Cell Lymphoma (DLBCL) was able to establish clustering of a specific oncogenic subpopulation (19). The applications of spatial modelling can also be extended to infer cell-cell communication through graph neural networks (20).

Studying the cell phenotypes from multiplex data has helped achieve a deeper understanding of the TME, with significant developments being made in single cell resolution of multiple antigens (21, 22). In turn, this has expanded the scope of spatial statistics that can be applied to digitized images from lo-plex and hi-plex platforms, thereby finding applications in different cancers (23, 24). Several spatial studies have deepened our appreciation and significance of intratumoral heterogeneity and its contribution in disease progression (25–27). In addition to examining the overall spatial landscape of tumors, identifying the spatiotemporal distribution of immune subsets is crucial to deconvolute the spatial niches within a tissue section (28, 29). Functional and spatial characterization of cell types through an integrative approach can also give leads on potential intercellular signals in the TME (30). There are a number of studies on the TME that use a combination of spatial modelling approaches (31, 32) to demonstrate how the malignant cells modulate the disease (33, 34).

A number of sophisticated tools that provide measures of the cellular heterogeneity and tumor infiltration have been developed to reveal features of the immune organization in tissues (35–37). The geospatial toolbox, spatstat (38), provides a range of mathematical functions that can be applied to examine cell-cell clustering patterns, while acknowledging its limitations in addressing the diversity of interactions within a ROI. There are different modules that allow the visualization of spatial expression data (39) and the resolving of cell-types at single cell level from pixel analysis of multiplex images (40). Additionally, tools have been developed to quantify spatial interactions between cell types for different imaging platforms (36, 41). Studies on the architecture of the spatial transcriptome have also revealed features such as the tertiary lymphoid structures (TLS) (42), which are among the major origins of tumor infiltrating lymphocytes (TILs) and which drive antitumor immunity (43). TLS can also be identified using methods applied to digitized histopathology images (44, 45).

There are evidences in literature to support the importance of relative organization and interaction among the cells in driving response (13, 46, 47). The use of mathematical clustering or inhibition models can reveal the effect of one observation on another (second-order effects); eg: the influence of the malignant cells on the distribution of immune cells in the TME. The distribution of immune cells relative to malignant cells and their population in tumor rich clusters can indicate which patients respond to checkpoint therapy. In this review article we discuss a number of tools and mathematical models that can be applied to the two-dimensional representation of the mIF images as spatial point patterns. The functions discussed here provide measures of clustering and have been developed decades earlier for studying geospatial data and ecological niches, as mentioned above. The application of these methods in a context specific manner to the TME is crucial in deriving insights that can advance clinical care and patient driven treatment. A number of reviews have discussed spatial methodologies for studying the TME and for assessing the intra tumor heterogeneity (25, 48). The application of mathematical functions to spatial point patterns or proceses requires single cell resolution from the pathologist annotated or the software determined nuclear co-expression with other biomarkers. The robustness of these spatial methods is dependent on the single-cell resolution of the imaging or microscopy techniques. The PhenoCycler-Fusion, multiplexed single-cell *in situ* profiling and multiplex immunofluorescence approaches can resolve single-cell expression of proteins. However, identifying two interacting cells demonstrating cell-cell contact is still a challenge. Spatial processes have been applied to regions examining elevation and rainfall, hence this approach can be extended to study the 3D neighborhood if there is addition information on tissue features. The composition of the TME in terms of different immune cells and soluble factors are well studied, however, examining the spatial neighborhood requires the cell coordinates to be accurately determined which can be a challenge with soluble factors or extracellular signals.

Here, we discuss ways to interrogate the mIF data and obtain local and global features from the tumor-immune relationships within the TME using different existing methods. We then interpret the findings from the methods and discuss their correlation with the observed spatial organization of the different cell phenotypes.

## Methods

### Spatial point patterns from mIF images

**Figure 1:**
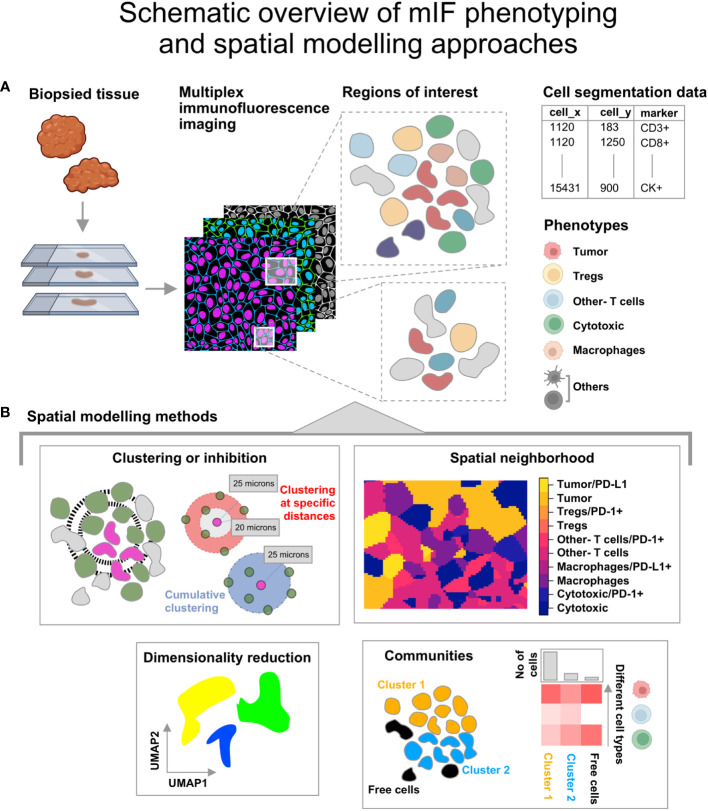
Illustration describing the steps from tissue collection to spatial analysis of multiplex immunofluorescence (mIF) images. **(A)** The biopsied tissue stained with the panel biomarkers is used to obtain cell segmentation data with marker expression for regions of interest. The co-expression for the listed phenotypes at single cell level is obtained. The icons used here are from Biorender.com. **(B)** Spatial modelling methods applied to the Tumor Micro Environment. -Clustering or Inhibition: This is measured using mathematical functions (Nearest Neighbor-G function or pair correlation function). -Spatial neighborhood: This is determined for each ROI by computing the spatially varying probability of cell phenotypes for each pixel grid. -Communities: Clusters of cells identified from hierarchical clustering based on Euclidean distance and the minimum cluster size. - Dimensionality reduction using graph based approaches that retain the topology of the data.

Any two-dimensional distribution of points that represent geographical features, species or cells can be used to generate spatial point patterns which are defined by a boundary or spatial window. Lo-plex images such as H&Es as well as images obtained from mIF and PhenoCycler-Fusion can be used to generate the point patterns after extracting the coordinates and marker information from the segmentation data, as shown in **Figure 1A**. The point patterns are amenable for further spatial analysis using mathematical functions available in the *spatstat* toolbox. An overview of the spatial modelling approaches discussed in this article are shown in **Figure 1B**.

**Figure 2:**
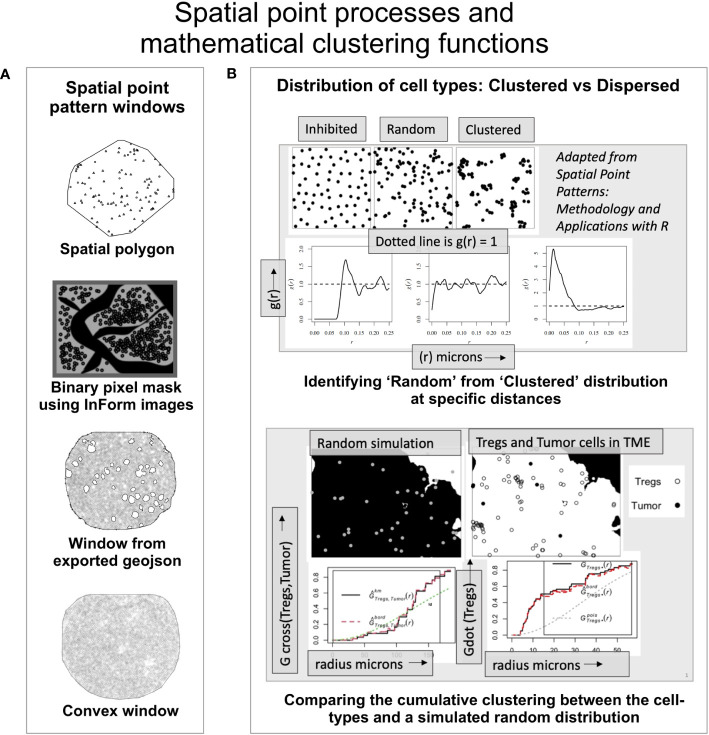
An overview of the spatial mathematical functions and point patterns. **(A)** Spatial point pattern windows. -Spatial polygon – A geometric shape demarcating the data (adapted from spatstat). -Binary pixel mask- generated by the inForm software from source images (adapted from a TME). -Window from exported geojson- Spatial window was built using the exported geojson annotations from QuPath using RGB mask image overlaid with the coordinates. -Convexhull – A convex window available in spatstat that can be used for circular TMAs. **(B)** (Upper panel) - The ordered, random and clustered distributions can be estimated at specific radius intervals using the pair correlation function *g(r)*. Shown below are the corresponding *g(r)* plots as a function of distance for the each distribution (above). (Lower panel) shows the distribution of a random process in the TME and the observed distribution of ‘Tregs’ and ‘Tumor’ cells. The red dashed line indicates edge corrected nearest neighbor G- function values for the observed data and compared with the random distribution (dotted line).

The point pattern windows can be spatial polygons, a non-intersecting geometric shape that defines the boundary of the points (**Figure 2A**). The InForm software generates a binary pixel mask from the exported images (i.e, composite and component images); resulting in a rectangular window enclosing the ROI with a binary mask as shown in **Figure 2A**. Alternately, the tissue microarray (TMA) coordinates from the csv file can be overlaid on the RGB images (with or without the tissue segmentation mask and biomarker channels). Using QuPath (49) the pixel classifier can be trained to generate the geojson annotations i.e. tissue boundaries of the ROI, which can be imported into an R script to generate the spatial window. With this approach, regions such as blood vessels, necrotic areas etc., can be excluded as shown in **Figure 2A**. For the circular TMAs, the *convexhull* function from *spatstat* is a straightforward choice. It produces a circular boundary around the points to generate the spatial window (**Figure 2A**).

### Pair correlation function

The empirical distribution of cell phenotypes in the image can appear clustered or dispersed, however this cannot be quantifiably assessed without robust spatial clustering functions. In **Figure 2B** (upper panel), the spatial distribution among inhibited, random, and clustered populations is illustrated with an example. Shown below are the pair correlation function - *g(r)* plots for the three spatial patterns respectively. The *pcf* function (from *spatstat*) allows the user an easier way to examine spatial clustering at different intervals of radii. The pcf function computes g(r) (i.e. *K*′(*r*)​/2*πr*). *K*′(*r*)​ is a derevative of the Ripley’s K function (18). For a random distribution i.e. a Poisson simulated from the underlying distribution, the *g(r)* values lie close to 1, while an inhibited population is more dispersed than a random distribution with *g(r)* < 1. A clustered population would imply points are closer than in a random distribution and consequently with most values of *g(r) >* 1, as shown in **Figure 2B**. This function can be used to quantitatively examine clustering within and between different cell populations or phenotypes at different values of *r*.

### Nearest neighbor G-function

The nearest neighbor *G*-function (*Gest* from *spatstat.core*) provides a cumulative distribution of points from the same (*Gdot*) or different cell phenotypes (*Gcross*). It examines the deviation of the empirical data from a theoretical curve which is generated from a random distribution of the cell phenotypes in the ROI. The assumption made for the examples discussed here is that they represent a homogeneous stationary process. A homogeneous point pattern has a uniform distribution throughout the ROI. The random simulation of these points is generated using the ‘intensity’ (number of points per unit area) and edge correction is applied to the point pattern. In **Figure 2B** (lower panel), through an illustration we have shown a comparison between the random simulation of the ‘Tregs’ and ‘Tumor’ with the observed distribution of these cells in an image. The relative clustering between ‘Tregs and ‘Tumor’ cells (i.e. Gcross(Tumor/Tregs)) is observed only at higher distances (red dotted line). The clustering within the ‘Tregs’ (i.e. Gdot(Tregs)) is significantly higher (red dotted line) than the random distribution of the population (dotted grey line).

### Neighborhoods based on spatially varying probability

The *relrisk* function in the spatstat toolbox (*relrisk.ppp* from *spatstat.core*) calculates the spatially varying probability of each marked point i.e. cell phenotype in the point pattern using a non-parametric estimate. Essentially, it calculates the probability that a point at a specific location belongs to a cell phenotype *j.* This calculation is extended over every spatial location for each pixel in the grid and a list of pixel images for each cell type is generated. The estimation is performed by Nadajara-Watson type kernel smoothing. The user can also specify numerical weights for the points of a specific cell-type. This feature may be useful if the cells are having different expression levels which can be used as weights.

### Cell communities based on hierarchical clustering

The point processes and functions cannot reveal local intratumoral heterogeneity in terms of cell neighborhoods composed of different cell populations. The clustering (using *pcf* and *Gest*) between cell-types is computed by examining every cell of type *a* with another cell of type *b* or the same cell-type. The differences in the immune-tumor interactions at the tumor boundary *vs.* the stroma are not taken into consideration through these pairwise clustering functions. The SPIAT library (35) allows the users to examine the multiplex images from different platforms and has a number of functions that can be applied for spatial analysis of the TME. The function *identify_neighborhoods* can group cells into clusters based on Euclidean distance and identify the relative percentage composition of each phenotype within these clusters. The hierarchical clustering is based on the user defined minimum neighborhood size and interaction radius. This method can lead to identifying dense networks of interacting cells or the overall immune composition in the tumor or stroma. The input for SPIAT is a spatial experiment (spe) object which is used routinely for storing spatial- omics data from different platforms and is an R/Bioconductor S4 class. The mIF data (*xy* coordinates, cell Id and phenotypes etc.) can be stored as an spe object in order to apply these functions.

### Minimum or average pairwise nearest neighbor distances

The SPIAT toolbox provides functions that calculate the nearest neighbor distances between the different cell populations using the *negDistMat* function. This does not generate a full distance matrix and instead uses a rectangular similarity matrix from a subset of samples for the distance-based calculation. For the examples discussed in this article the *minimum* and *pairwise* distances between cell-types is measured for the cells both within clusters and the cells that are ‘Free’. This can help understand cell interaction behavior within different regions of the tumor and stroma. The minimum nearest neighbor distances (from SPIAT) are computed using a *kd-tree* approach to identify approximate nearest neighbors for each cell-type in the dataset.

### Identifying bordering cells for a reference cell type

The SPIAT toolbox includes a function to identify the cells bordering a reference cell type (eg: ‘Tumor’ cells). This identifies clustered groups of the reference cell phenotype and the boundary cells using *alphahull*, a derivative from the *convexhull* approach. The programming for *alphahull* is based on the duality between the Voronoi diagram and Delaunay triangulation. The bordering cells are identified based on their occurrence if they are found on the *alphahull*. The arc or rim of the alphahull separates the cells that are ‘Outside’ from ‘Inside’ the reference cell cluster. The number of cells on the rim or *alphahull* constituting the ‘Border’ are significantly smaller than the cells labelled ‘Outside’ or ‘Inside’. This function is useful in identifying tumor bordering cells and the relative composition of immune cells that are ‘Inside’ or ‘Outside’ the TME.

### High dimensionality reduction

The segmentation data for mIF images contains spatial *xy* coordinates and biomarker information from which the phenotypes (i.e. features or labels) can be obtained. PCA (Principal Component analysis) (50) factorizes the data unlike UMAP (Uniform Manifold Approximation and Projection) which uses the neighbor graph approach and attempts to find such a graph in lower dimensions of the data (51). The UMAP builds a graph by approximating the shape of the data by connecting the simplices. UMAP based workflows have been applied to imaging mass cytometry data but can also be used for understanding the geographical organization of cells and their interactions (52).

### Softwares and packages

Scripts and spatial analysis codes for the examples discussed here were written in RStudio using R version 4.2.0. The spatial toolbox- spatstat v. 3.0.6 was used for clustering analysis. Phenoptr package (v. 0.3.2) was used for consolidating segmentation data. Community identification and border cell identification scripts were obtained and modified from the SPIAT (v. 1.0.4) github. A script in python (version 3) jupyter notebook was written to identify single cell expression of different biomarkers to determine phenotypes from the cell segmentation data generated by InForm.

## Results

### Spatial point patterns and phenotyping cell populations

The examples discussed in this article are point pattern representations of mIF images obtained from a malignant pleural mesothelioma study. A preliminary spatial analysis on this cohort by Parra et al. (53) has shown conclusive evidence of immune infiltration in these tumors by assessing 10 regions of interest (ROI) in each surgically resected case. The cell segmentation data for each biomarker in the panel (CD3, CD8, CD68, CK, PD-1, PD-L1, KI67 and FOXP3) was used to consolidate the co-expression data at single-cell level using a python script (Jupyter Notebook). Point patterns were generated for each of the samples using the *phenoptr* package. A rectangular spatial window was generated using the min- and max- of the cell *xy* coordinates. Phenotypes (Tumor - CK+, Cytotoxic - CD3+CD8+, Tregs - CD3+FOXP3+, Macrophages - CD68+, Other- T cells – CD3+CD8-) were characterized using the lineage and functional markers. The phenotype ‘Others’ was subset from the remaining cell populations (**Figure 1A**). For this article, five examples were identified from the cohort based on their distinctive immune-tumor landscapes. The spatial analysis methods applied to the TMEs discussed in this article are illustrated in **Figure 1B**. The spatial windows demarcating the point pattern boundaries can be generated from different TMAs and are shown in **Figure 2A**. **Figure 2B** (upper panel) shows the plots corresponding to different clustering patterns. **Figure 2B** (lower panel) describes the spatial distribution of ‘Tregs’ and ‘Tumor’ cells in an example TME and the corresponding nearest neighbor *G*-function curves for them. As can be seen, the clustering between ‘Tumor-Tregs’ is poor while clustering between ‘Tregs-Tregs’ is comparatively stronger.

### Spatial distribution of cells by phenotype

**Figure 3:**
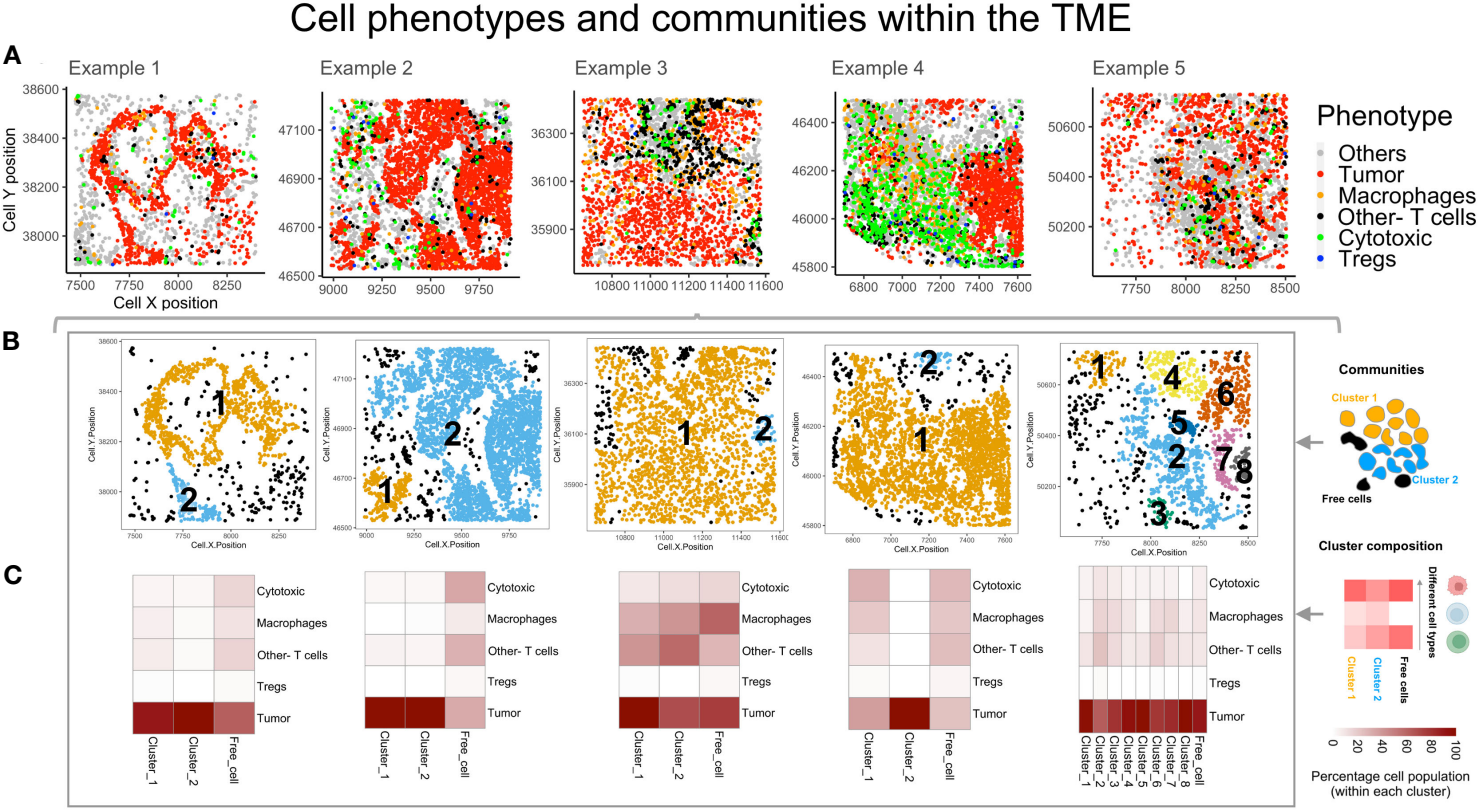
Spatial organization of cells by phenotypes and identifying communities. **(A)** The different cell phenotypes are plotted for five example TMEs: Tumor (red), Th (black), Cytotoxic(green), Tregs (blue) and Others (grey). **(B)** Cell communities are identified based on hierarchical clustering and Euclidean distance. The different colors indicate unique clusters and indicated within the plot are the cluster numbers. **(C)** Heatmaps for the clusters (above) indicate the relative percentage composition of different cell phenotypes within each cluster.

The cell coordinates and phenotypes, obtained from the consolidated segmentation data, were used to generate a spatial experiment (spe) object containing cell ID, phenotype and *xy* positions. The SPIAT toolbox functions were used to plot the cells by phenotype, as shown in **Figure 3A**, with spatial positions indicated on *x* and *y* axes. This provides a visualization of the manner in which the immune cells are distributed vis-à-vis the tumor cells. From these plots, the differences in the organization of immune and tumor compartments can be observed. For eg: the clustering among the ‘Tumor’ cell phenotype in examples 2 and 3 (**Figure 3A**) appears to be higher than in the other examples (1 and 5). The immune cell phenotypes (Tregs, Other- Tcells and Macrophages) are more segregated from the tumor rich region in example 3 than example 5 (**Figure 3A**). The performance of these methods correlates with the selection and quality of the ROI. To compare ‘Tumor’ clustering of the examples 1-3 and 5 with 4, the regions should be similar i.e. tissue taken either from tumor core vs. tumor-stroma boundary. The size and region of the tissue taken for mIF will determine if the inferences from spatial analysis can be generalized for a patient. To identify the tumor-immune interaction pattern for a patient, one should select multiple ROIs from different regions (infiltrating edge, tumor core, normal tissue boundary etc) and assess potential differences in cell behavior/interaction across these regions.

These plots provide the background to apply context dependent spatial mathematical functions and to obtain quantifiable measures of differences in the TME. These observations may correlate with the data, while also capturing differences that cannot be obtained from global distribution of cell populations.

### Cell communities in the intratumoral regions

Using the data exported as spe objects and the SPIAT toolbox functions, cell communities within each of the TMEs (**Figure 3A**) were identified through hierarchical clustering, Euclidean distance of 25 microns and minimum neighborhood size of 25 cells. These were termed as ‘Clusters’ and are numbered, as shown, in different colors (**Figure 3B**). The algorithm designates those cells that are not within the interaction radius specified and whose size < 25 cells as ‘Free cells’ (shown in black **Figure 3B**). This feature was useful in identifying different communities in the TME, constituting different intratumoral interacting groups of cells. This is useful in identifying interactions that could suggest a favorable response to immunotherapy, as immune cells interact with tumor nests in a contact dependent manner. The number of cells and composition within the largest clusters in each example (right to left – Cluster 1, 2, 1, 1 and 2) were distinct. The largest cluster in example 3 showed maximum immune infiltration (**Figure 3C**). The heatmaps show that the ‘Other- T cells’ cells and ‘Macrophages’ are the larger fraction of the immune cells. In example 4, a large cluster of immune and tumor cells which are segregated (in **Figure 3A**), is found with phenotypes ‘Other- T cells’, ‘Macrophages’ and ‘Tregs’ in comparable numbers. Within example 5, there are multiple clusters, with the largest having poor immune infiltration and a relatively small number of ‘Other- T cells’, ‘Macrophages’ and ‘Tregs’. Across all other clusters, the relative percentage composition of immune cells remains similar. Overall, among all the examples shown here, the ‘Tregs’ are the lowest population among the immune-tumor clusters and also among the ‘Free cells’.

### Clustering between tumor and immune cells – global and local

**Figure 4:**
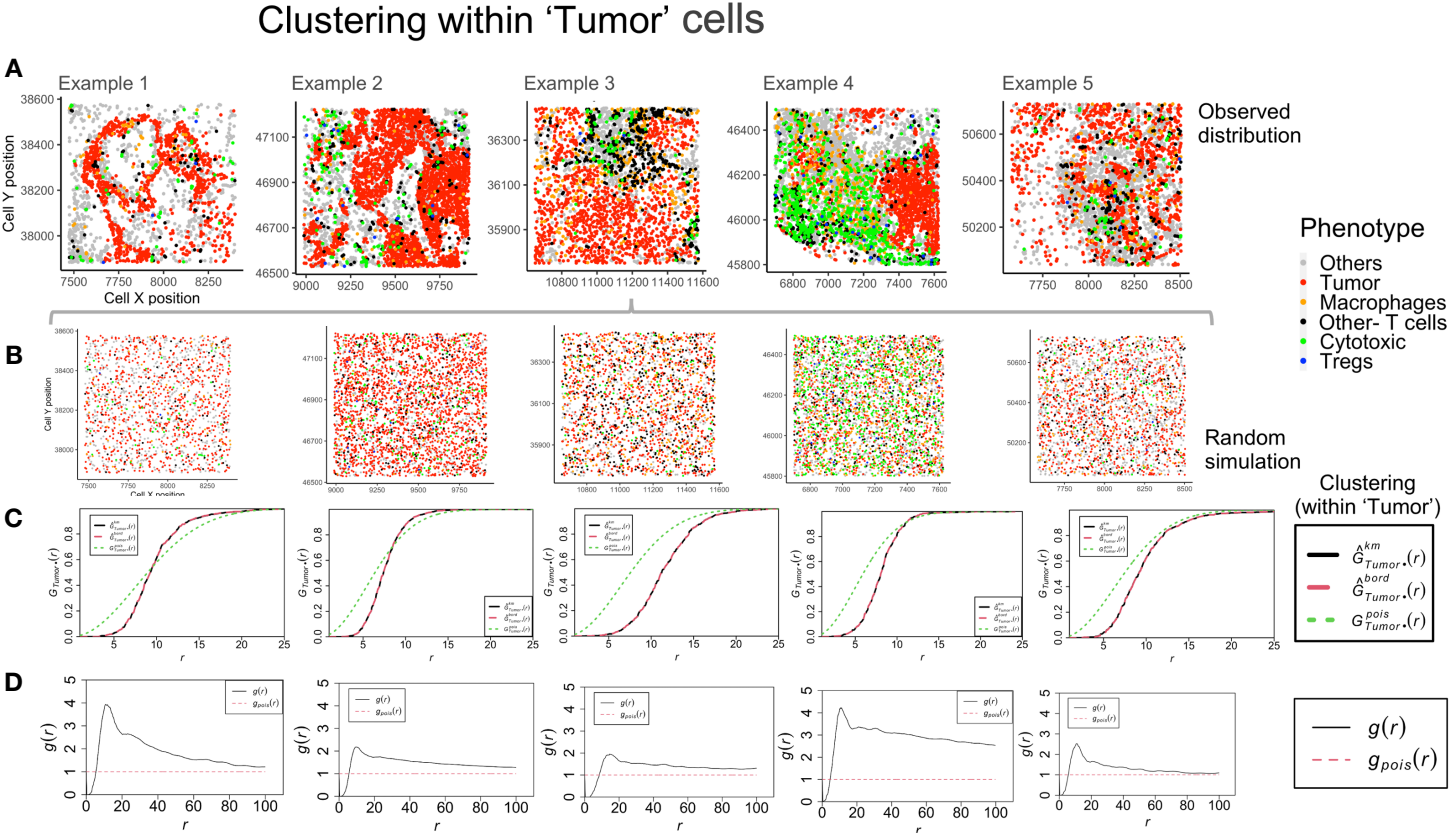
Clustering patterns within the example TMEs among the ‘Tumor’ cells. **(A)** The different TMEs and the cell phenotypes are plotted for the observed data. **(B)** A random point process distribution for each phenotype is generated and combined in each plot. **(C)** Nearest neighbor G- function clustering plots as a function of distance (r) for the ‘Tumor’ cells in the above examples. The edge corrected values are shown in dashed line (red) and the random distribution in dotted line (green). **(D)** Pair correlation function values *g(r)* as a function of distance (r) for the ‘Tumor’ cells is shown with the red dotted line indicating a random distribution and the black solid line indicating the observed values.

The global clustering among tumor and immune cells can be determined through the nearest neighbor *G*-function and the pair correlation function (*PCF*). The G-function can estimate the clustering aggregated over distance for multitype point patterns (between different cell-types) and within the same population. The cumulative distribution is compared with an underlying simulated random point pattern to find measurable indicators of clustering. For the TMEs shown in **Figure 4A**, a Poisson process was generated (*rpoispp*) for each image using the ‘intensity’ values for each phenotype. These processes were then combined using the *superimpose* function, as shown in **Figure 4B**. The number of cells of each phenotype in the ROI is termed ‘intensity’ and is used to generate the random point pattern distribution. Shown in **Figures 4C, D** are the plots derived from two mathematical functions that measure clustering at a global and a local scale, respectively. The Nearest neighbor *G*-function clustering between the ‘Tumor’-’Tumor’ phenotype (dashed red line in **Figure 4C**) shows that the clustering is weak in example 1-2. In the other examples, clustering is lesser than the random Poisson distribution (dotted green line in **Figure 4C**). This implies that the ‘Tumor’ cells are farther apart than would be seen if they were randomly distributed. However, in **Figure 4D**, the pair correlation function, which is a measure of clustering at definite radius intervals, finds g(r) values > 1 (solid black line in **Figure 4D**). This is a quantifiable measure of clustering in comparison to complete spatial randomness (dotted red line in **Figure 4D**). The plots for example 2 and 3 are similar, even though their TMEs (**Figure 4A**) appear visually distinct. The *PCF* plots for example 1 and 4 show the highest clustering among the ‘Tumor’ cells.

**Figure 5:**
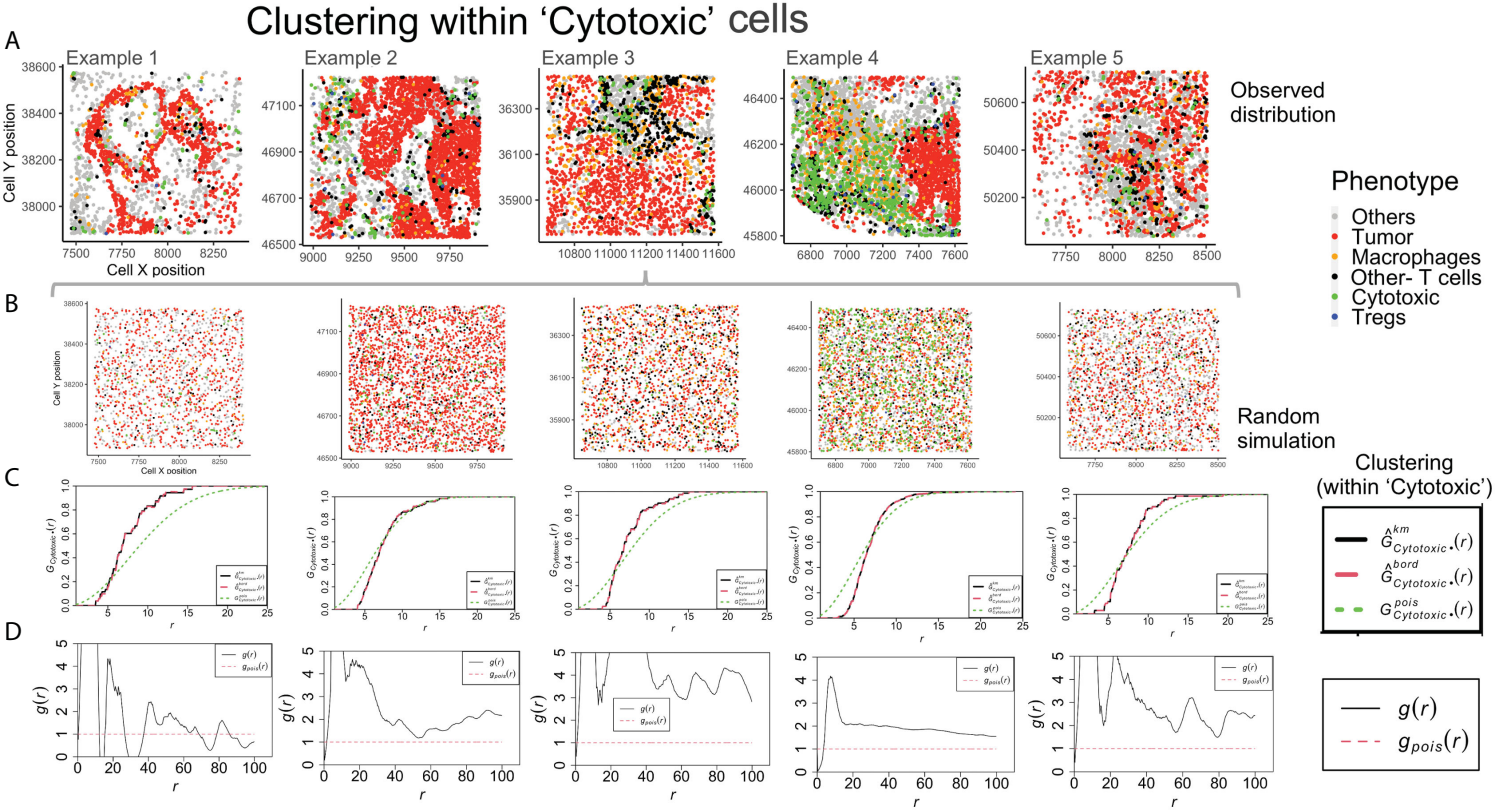
Clustering patterns within the example TMEs among the Cytotoxic cells. **(A)** The different TMEs and the cell phenotypes are plotted for the observed data. **(B)** A random point process distribution for each phenotype is generated and combined in each plot. **(C)** Nearest neighbor G- function clustering plots as a function of distance (r) for the ‘Cytotoxic’ cells in the above examples. The edge corrected values are shown in dashed line (red) and the random distribution in dotted line (green). **(D)** Pair correlation function values *g(r)* as a function of distance (r) for the ‘Cytotoxic’ cells is shown with the red dotted line indicating a random distribution and the black solid line indicating the observed values.

Example 4 shows a distinct neighborhood of ‘Cytotoxic’ cells in **Figure 5A**. However, the clustering between ‘Cytotoxic’-’Cytotoxic’ cells measured using *G*- function is highest in example 1, lower in example 3 and 5 and least in example 4. The answer to this unexpected outcome lies in the random distribution for example 4, as shown in **Figure 5B**. The high density of ‘Cytotoxic’ cells leads to no significant difference between complete spatial randomness and empirical distribution using *G*- function **Figure 5C**. Interestingly, we observe appreciable differences in the respective *PCF* plots for the ‘Cytotoxic’-’Cytotoxic’ cell clustering, as seen in **Figure 5D**. The highest clustering is seen in examples 2, 3 and 5, while the lowest is seen in example 4.

### Spatial neighborhoods based on probability

**Figure 6:**
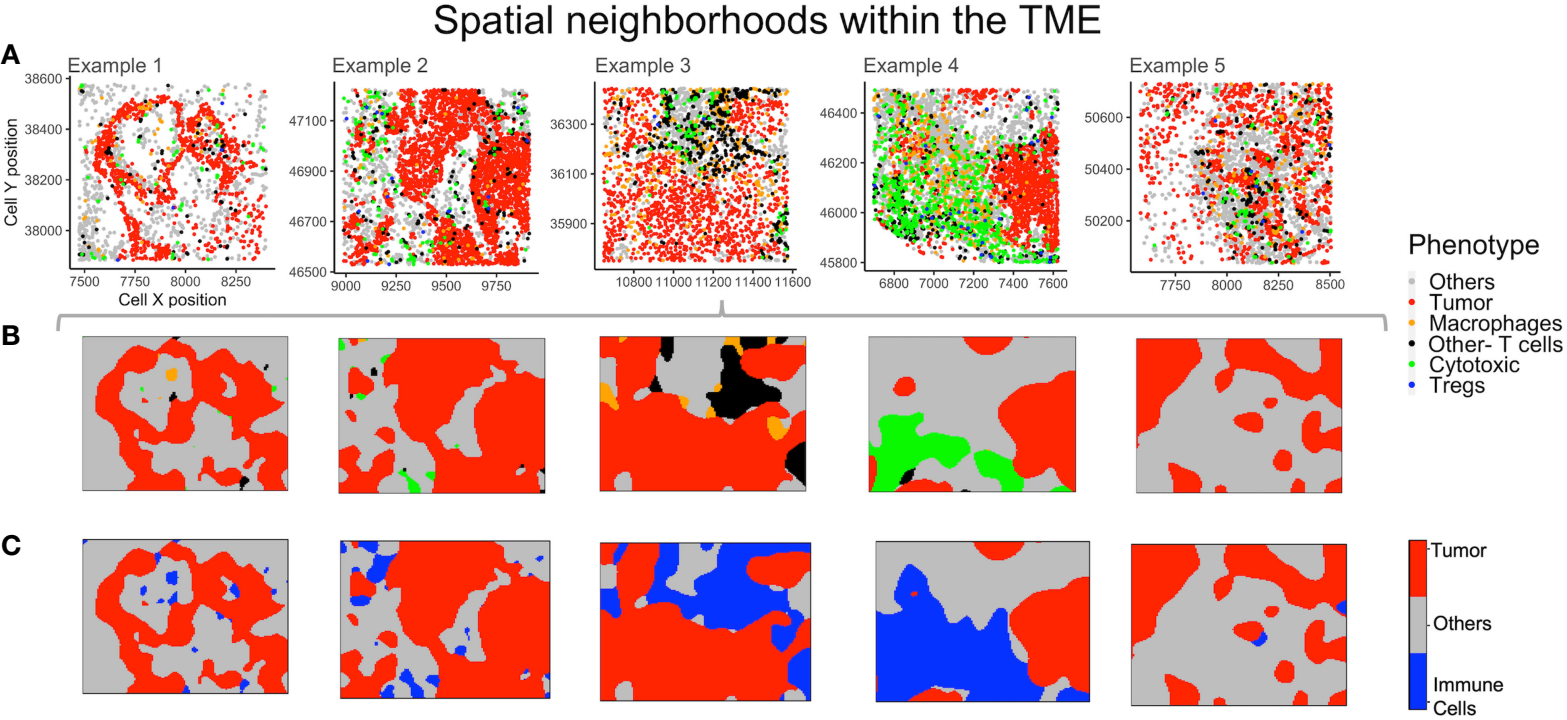
Neighborhoods obtained from the spatial probability of each phenotype. **(A)** Different TMEs and the cell phenotypes are shown for reference. **(B)** The spatially varying probability of each phenotype computed and plotted using the same color as the phenotypes for the above TMEs. **(C)** Aggregating immune cells to compute spatial neighborhoods for the phenotypes – ‘Others’, ‘Tumor’ and ‘Immune cells’ (shown in grey, red and blue respectively).

The non-parametric estimates of spatially varying probability for each phenotype were plotted to find neighborhoods in the example TMEs (**Figure 6A**). This method identifies the most probable phenotype for each grid in the point pattern, viz. for each mIF image, which can then be plotted. The use of phenotype ‘Others’ is helpful in increasing the accuracy of spatial neighborhood calculation, as the probability is calculated for every pixel grid in the image. In **Figure 6B**, the spatial neighborhoods for each phenotype are plotted and can be compared by cell phenotypes shown above (**Figure 6A**) in the same color. As ‘Others’ is a dominant phenotype in terms of abundance, we combined the three known immune cell phenotypes (Macrophages, Tregs and Other- T cells) into one category - “Immune cells”. The combined probability for all ‘Immune cells’ is plotted in **Figure 6C**. Examples 3 and 4 have distinctively higher immune population in comparison to examples 1, 2 and 5 (**Figure 6A**). From the spatial neighborhood plots (**Figure 6C**) it can be observed that the immune cells are more segregated in example 4 as compared to in 3.

### Nearest neighbor distances among ‘Free cells’ and ‘Clusters’

**Figure 7:**
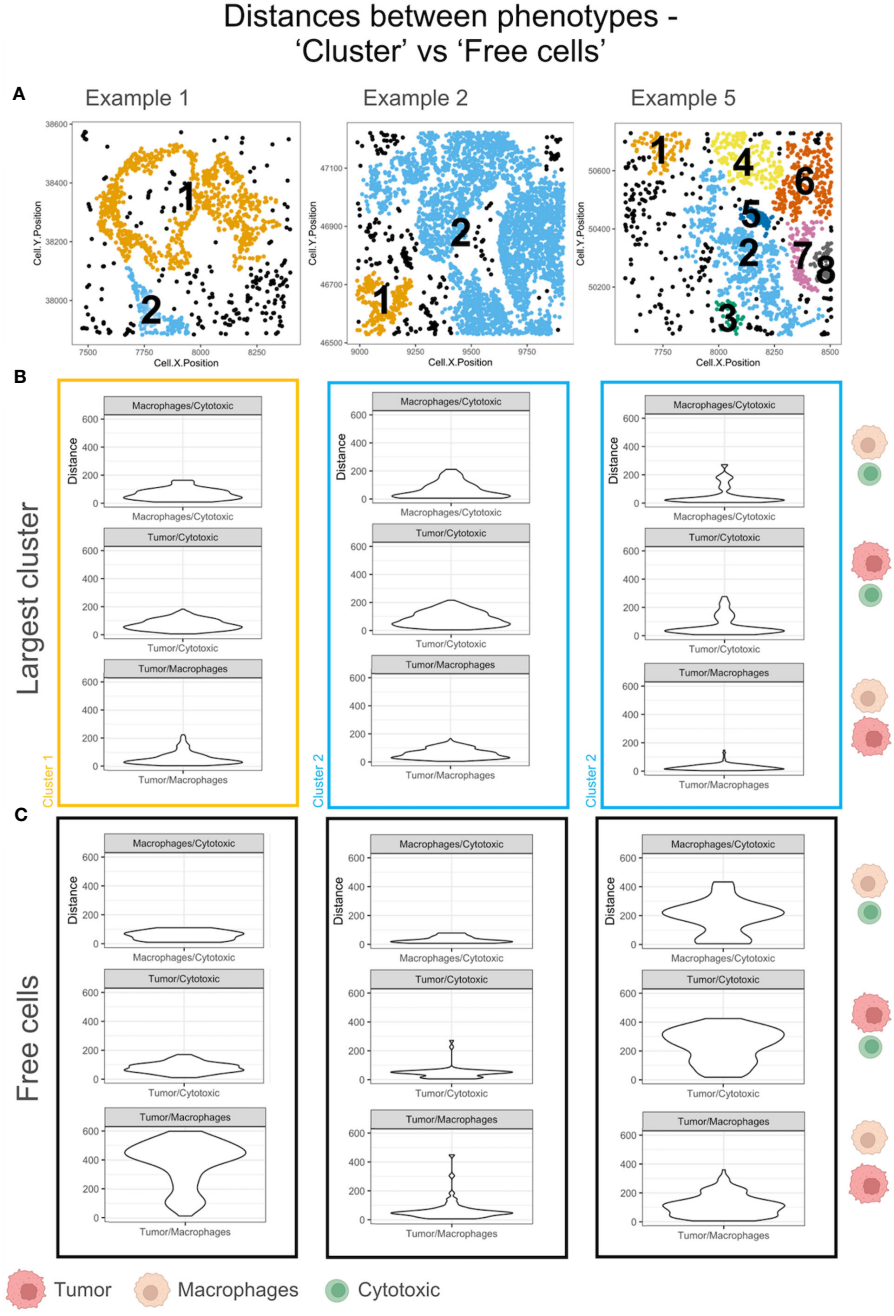
Minimum pairwise distances computed for the largest cluster and ‘Free cells’ in each TME. **(A)** Cell communities shown with unique clusters in colors and ‘Free cells’ shown in black. **(B)** The violin plots of the minimum pairwise distances between Macrophages/Cytotoxic, Tumor/Macrophages and Tumor/Cytotoxic cells for the largest cluster in A. The cluster color is used to outline the violin plot. The cell phenotypes examined per plot is indicated on the right with a schematic (legend: below figure). **(C)** The violin plots of the minimum pairwise distances between Macrophages/Cytotoxic, Tumor/Macrophages and Tumor/Cytotoxic cells for the Free cells in A. The cell phenotypes examined per plot is indicated on the right with a schematic (legend: below figure).

The ‘average pairwise distances’ and the ‘minimum distance’ functions in SPIAT are two measures of the interacting distances between cells of different phenotypes (eg: ‘Cytotoxic’ and ‘Tumor’ cells) that can be applied to the TMEs. Intratumoral heterogeneity has been found in most tumors. To illustrate this difference, we have compared the minimum distance within the largest cluster and the ‘Free cells’ in each TME. The phenotypes – ‘Macrophages’, ‘Tumor’ and ‘Cytotoxic’ – were selected for the distance measurements in examples with a sizeable population of ‘Free cells’ (**Figure 7A**). The minimum distances between ‘Macrophages’ and ‘Cytotoxic’ cells were appreciably different for example 5 in the largest cluster (shown in blue in **Figure 7B**), as compared to the ‘Free cells’ (**Figure 7C**). The ‘Cytotoxic’-’Tumor’ cell distances were different between the large cluster and ‘Free cells’ for examples 2 and 5 (both shown in blue). Differences in the ‘Macrophage’-’Tumor’ cell distances were the most distinct in the largest cluster of example 1(shown in orange), which is in agreement with the distribution of ‘Free cells’ in the TME (**Figure 7A**) that appear dispersed. There is an appreciable difference in the ‘Macrophage’-’Tumor’ cell distances for example 1 between the largest cluster and the ‘Free cells’ as well.The comparisons discussed above are representative and not an exhaustive comparison for all pairwise cell interactions of all example TMEs.

### Identifying cells bordering tumor regions

**Figure 8:**
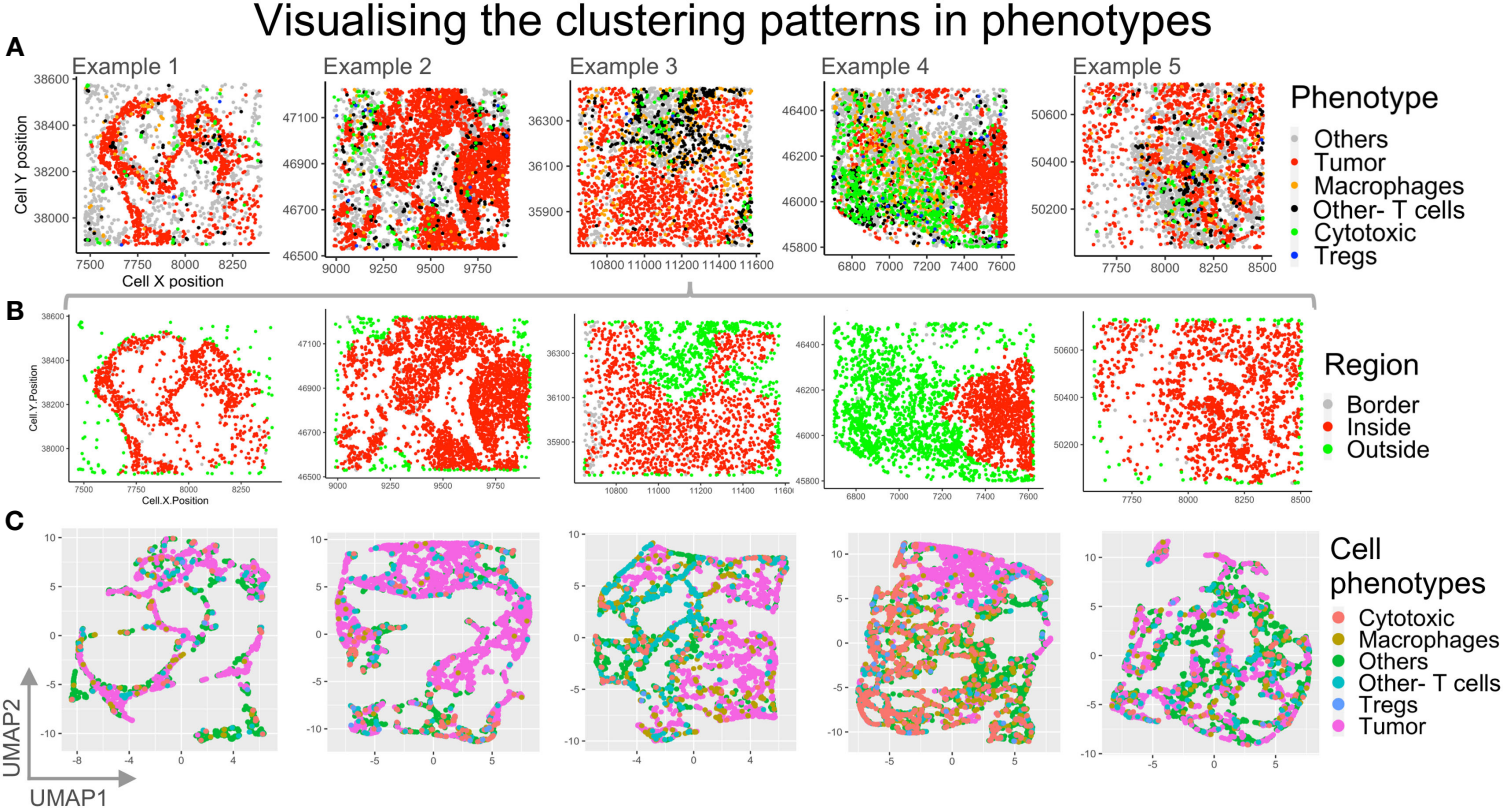
Visualizing different clustering patterns among the phenotypes. **(A)** Cell phenotypes identified are plotted for the five example TMEs: Others (grey), Tumor (red), macrophage (orange), other T cells (black), cytotoxic T cells (green), Tregs (blue). **(B)** The reference cell type (Tumor) bordering cells are identified using an approximation with border cells on the alphahull (in grey), cells inside tumor rich regions (in red) and cells outside the tumor rich regions (in green) plotted. **(C)** Dimensionality reduction using UMAP with spatial coordinates as features and phenotypes as labels. .

In order to identify if any cell types may be regionally associated with a reference cell type, phenotypes were identified and plotted (**Figure 8A**). The cells bordering a reference cell phenotype, eg: ‘Tumor’, were identified using a SPIAT function based on the *alphahull* function. For our examples, this translated to identifying tumor rich clusters and their bordering cells as well as cells lying outside the reference cell cluster (**Figure 8B**). Cells inside the reference cell cluster, i.e. the ‘Tumor’ rich regions, are shown in red. The cells outside are shown in green, while the bordering cells are shown in grey. The bordering cells were identified based on an approximation (*alphahull)*, with cells on the *alphahull* designated as ‘Border’ cells. Hence, the ‘Border’ cells are found on the rim or arc i.e. the *alphahull* and are consequently a small proportion in comparison to cells ‘Inside’ or ‘Outside’ the tumor rich clusters. The cells outside the alphahull are designated as ‘Outside’ and shown in green. The relative proportion of immune cells that are ‘Outside’ can be used to identify immune infiltration in the tumor region. Fine tuning the cluster size and *alphahull* parameters per region can lead to an accurate representation of the tumor rich clusters, as shown in **Figure 8B**. The ‘Others’ phenotype were excluded to understand the immune cell organization and if they were predominantly on the ‘Border’, ‘Outside’ or ‘Inside’ the tumor rich clusters. In **Figure 8B**, example 4 shows a clear demarcation between the ‘Inside’ (red) and ‘Outside’ (green) regions. Comparison with **Figure 8A** shows that the cell phenotypes corresponding to ‘Inside’ are mostly Tumors, while immune cells are ‘Outside’, for example 4. Also, in example 3, an immune rich region can be seen distinct from the tumor regions in **Figure 8A**. This separation is observed in the corresponding region in **Figure 8B** as well.

### High dimensional spatial analysis of mIF images

High dimensionality reduction based on Uniform Manifold Approximation and Projection (UMAP) is shown in **Figure 8C**. For each of our examples, cell *xy* coordinates were used as features, while phenotypes were used as labels, for UMAP clustering. Clustering patterns of cell phenotypes represent the topology of data, which, for the mIF images, is the spatial distribution of the cells in the TME. The high dimensional reduction is applied routinely to identify patterns of similar gene or protein expression from flow cytometry and RNAseq data. This method can be applied to spatial data from mIF to determine spatial grouping of different cell phenotypes. In this case we can appreciate through the UMAP plots for example 3 and 4 that the ‘Tumor’ cell phenotype (pink) clusters are distinct from the immune cell clusters.

## Discussion

The emergence of multiplex imaging techniques has enabled interrogating spatial organization of different cell phenotypes in the TME and has simultaneously incentivized the development of computational methods to model this data (21, 54–57). The spatial analysis of mIF images provides preliminary insights into the immune-tumor interactions, allowing visualization and quantification of immune subsets. This is useful to identify the infiltrating immune cell populations (such as PD-1+ T cells) and the tumor cells expressing the PD-L1, thus improving prediction of response to checkpoint therapy (58). In this context, the spatially varying probabilities function is useful in distinguishing the TME within the five examples discussed above. The immune cells in example 4 are largely outside the tumor core as opposed to example 3 which shows higher immune infiltration. For hot tumors the *Gcross* function can be used to measure if the immune infiltration significantly higher than if the cells were randomly dispersed (13).

However, the disadvantage of modelling the TME using mIF is that the patterns of clustering or inhibition are derived from a subset of the total resident immune populations. Consequently, the contribution of other immune features, through association, in driving a clinical outcome are not obtained (59). Also, mIF tissue regions are smaller, which does not provide sufficient information about spatial heterogeneity of the cell populations in the tumor-stroma region. As the cellular locations within mIF ROIs unlike geographical features are dynamic, therefore the interpretations from mathematical functions should be generalized after examining a large number of regions. Hence, it is important that clustering functions (*pcf and Gdot/Gcross*) are applied to multiple patient ROIs to make clinically relevant spatial findings. The ROI selection and/or use of tissue microarrays as opposed to whole slide analysis is an important metric upstream of applying these analysis strategies. In this study, we originally selected 10 ROIs for analysis with the intent of expanding our understanding of the heterogeneity of the TME (53). Recent work from Sun et al., has shown that selection of 5 ROIs is able to reasonably recapitulate the TME in non-small cell lung cancer whole slide sections (60). The dependence on tissue availability and fluorescent probes that deviate from pathology standards are other technical limitations with mIF. The key consideration for the application of spatial methods and mathematical functions to the multiplex images is selecting ROIs representative of the specific cancer TME and relevant to the hypothesis and scope of the study.

Extending spatial modelling approaches to high dimensional platforms with pan immune markers can enable a deeper understanding of the different myeloid populations in driving the antitumor immune response. In this article, we have explored the spatial models relevant to study the immune-tumor landscape from mIF images. However, spatial modelling approaches applied to data from high dimensional imaging platforms, such as the PhenoCycler-Fusion, would give an in depth understanding of the different players from the immune system in the TME specific to a cancer type (61–64). This will also improve the above discussed mathematical functions’ performance and accuracy, which is reciprocally related to the sparseness and quality of the input data.

## Data availability statement

Data and materials can be made available upon request to the corresponding authors.

## Ethics statement

Patients provided written informed consent based on the Declaration of Helsinki principles. Tissue from surgical resections was used under a protocol approved by the University of Texas MD Anderson Cancer Center’s Institutional Review Board.

## Author contributions

GK: data curation, formal analysis, investigation, methodology, writing – original draft, conceptualization. RP: writing – review & editing, data curation, resources. EP: writing – review & editing, data curation, methodology, resources. KK: conceptualization, supervision, writing – review & editing. CH: conceptualization, funding acquisition, supervision, writing – review & editing.
